# Polyphenols and Health Benefits: Volume I

**DOI:** 10.3390/foods13081221

**Published:** 2024-04-17

**Authors:** Joyce Trujillo, Victoria Ramírez

**Affiliations:** 1 Consejo Nacional de Humanidades, Ciencias y Tecnologías (CONAHCyT), San Luis Potosí 78216, Mexico; daniela.trujillo@ipicyt.edu.mx; 2 División de Materiales avanzados, Instituto Potosino de Investigación Científica y Tecnológica (DMA-IPICYT), San Luis Potosí 78216, Mexico; 3 Departamento de Cirugía Experimental, Instituto Nacional de Ciencias Médicas y Nutrición Salvador Zubirán, Tlalpan, Ciudad de México 14080, Mexico

Natural polyphenols are functional and bioactive substances widely present in plant-based sources such as fruits, vegetables, and other food items. Polyphenols may be considered non-essential nutrients, but they have numerous health advantages in the treatment of cardiovascular diseases, cancer, diabetes, degenerative diseases, aging-related conditions, and infections [1,2]. Polyphenols can be divided into four groups based on their chemical structure: flavonoids, phenolic acids, lignans, and stilbenes [3].

In this context, this Special Issue, “Polyphenols and Health Benefits,” features two research papers and three reviews. These papers and reviews highlight the importance of dietary interventions and the use of natural foods rich in polyphenols, which may contribute to the management of various health conditions (e.g., antioxidant, antitumoral, antibacterial, anti-inflammatory, hypoglycemic, and hypolipidemic effects across several diseases, including cancer, obesity, hyperuricemia, diabetes mellitus, stroke, dyslipidemia, cardiovascular diseases, and renal damage), highlighting their potential as alternative or complementary therapeutic approaches. Notably, this Special Issue explores polyphenol content in citrus fruits, seed extracts, agricultural grasslands, and *Curcuma longa*. These manuscripts describe the primary functional and bioactive metabolites associated with polyphenols and validate their functional activities.

The fruit of the date palm (*Phoenix dactylifera* L.) is an essential crop in the arid and semi-arid regions of the world, yet few studies have been published on date palm seeds [4]. In this Special Issue (Contribution 1), Swaidan et al. characterized the bioactive compounds in aqueous date seed extracts from six varieties. The phenolic content includes the following substances, from highest to lowest concentration: caffeic acid derivative, proanthocyanidin trimers, dimers, tetramers, catechin, and quercetin. These polyphenols were found in greater quantities in three of six varieties studied. The powerful antioxidant, antibacterial, and anticancer activities were associated with the content of total polyphenols. Also, they found antibacterial activity in Gram-positive but not Gram-negative strains (principally in two varieties). Interestingly, they observed that polyphenol-rich extract reduces cell viability in colon and lung cancer cells, providing evidence for the potential use of polyphenols in medicine.

In addition, Verhulst et al. (Contribution 2) compiled evidence on four grassland crops (*Lolium perenne* L., *Cichorium intybus* L., *Plantago lanceolata* L., *Trifolium pratense* L.) and their potential contributions as an alternative source of proteins from their aerial parts [5], contributing to food security by their lack of toxic effects. Also, they point out that these plants have a high content of polyphenols but are underutilized; their addition in regular diets may increase the proportion of nutraceutical value in food intake. Currently, some of these grasslands are utilized for high-quality forage in livestock feed, which improves milk quality, and they were integrated into agricultural practices due to their nitrogen fixation capacity.

All evaluated grassland crops contained significant quantities of phenolic acids (such as caffeic, chicoric, chlorogenic, and verbascoside), flavonoids (including quercetin, rutin, luteolin, and catechin), and isoflavones (such as daidzein, genistein, apigenin, formononetin, and biochanin A). This review focuses explicitly on demonstrating their antioxidant properties (observed in all grasslands studied) and their potential as anticancer, antidiabetic, and antiobesogenic agents (*C. intybus* L., *P. lanceolata* L., *T. pratense* L.). These crops’ phytochemical composition and bioactivity are significant because they can be utilized as potential pharmacological and nutraceutical contributions to improve human health.

In this Special Issue, three contributions focused on the relationship between the bioactive compounds in foods and their healthy beneficial effects. The first examines diet-related compounds’ antihyperglycemic effects (Contribution 3). The second contribution delves into the effects of curcuminoids on dyslipidemia in chronic kidney disease (CKD), which is linked to cardiovascular damage (Contribution 4). Lastly, the third explores the effects of hesperitin–copper II, a complex form of a flavonoid from citrus fruits that was previously enhanced by adding a metal ion. It aims to improve its physicochemical properties, such as solubility, stability, and bioavailability (Contribution 5).

Alfahel et al. (Contribution 3) explore the impact of bioactive compounds found in foods [6], such as phenolic acids (e.g., rosmarinic and caffeic acids) and flavonoids (e.g., hesperetin, resveratrol, rutin, quercetin, lutein, genistein, phloretin, apigenin, daidzein, catechins, and capsaicinoids), on chronic disease prevention. Over the past decade, experimental and clinical studies have revealed their potential in mitigating conditions like obesity, diabetes, cancer, and cardiovascular disorders.

Polyphenols act through various mechanisms, including combating oxidative stress, modulating gene expression, and regulating glucose metabolism. They also benefit gastrointestinal health, gut microbiota, pancreatic–endocrine functions, and weight management. Curcumin may have potential use in this last area. Moreover, flavonoids have been shown to regulate glucose metabolism and inhibit cancer cell proliferation. The chronic administration of anthocyanins or genistein in diabetic patients or women with metabolic syndrome reduces dyslipidemia and offers cardiovascular protection.

Further continuing this line of research, Ceja-Galicia et al. (Contribution 4) emphasize the importance of using curcuminoids to handle dyslipidemia in CKD, a prevalent condition worldwide. Dyslipidemia significantly increases cardiovascular mortality in this population, and conventional pharmacological treatments often come with side effects [7]. Thus, exploring alternatives like curcuminoids, which may have minimal side effects, becomes imperative.

The authors explore the pathophysiological aspects of dyslipidemia and its correlation with CKD, highlighting mechanisms such as oxidative stress, inflammation, fibrosis, and metabolic reprogramming. They discuss the bioavailability and effects of curcuminoids in both experimental models and clinical studies, demonstrating their efficacy in addressing dyslipidemia and associated cardiovascular damage. This includes modulating genes involved in metabolic pathways, improving lipid balance, and enhancing liver and kidney function (including reduced oxidative stress and improved mitochondrial function in both tissues).

Lastly, Peng et al. (Contribution 5) demonstrate the impact of hesperitin–copper II on hyperuricemia and renal inflammation, stemming from chronic elevations in uric acid levels due to purine metabolism disorder, potentially affecting vital organs such as the liver and kidneys, leading to complications like fibrosis [8]. This study evaluates the oral administration of hesperitin in a murine hyperuricemia experimental model, using biochemical, histopathological, and molecular analyses to reveal that hesperitin improved uric acid, creatinine, and BUN levels, preserved renal architecture, reduced oxidative stress by modulating antioxidant activity, and alleviated renal inflammation via a specific inflammatory pathway (NLRP3 nucleotide-binding domain, leucine-rich-containing family, pyrin domain-containing-3) that includes interleukins 1 and 6, as well as transforming growth factor beta (TGF-β) and tumor necrosis factor-alpha (TNF-α). The authors link these findings to enhanced enzyme activities responsible for uric acid catabolism and the modulation of urate transporters.

All this evidence shows that bioactive compounds, such as polyphenols, have potential use in dietary supplements, nutraceuticals, or medical therapy. However, further clinical trials are necessary to elucidate the pharmacokinetics and pharmacodynamics of several bioactive compounds for clinical application in managing chronic disease and their potential applications in pharmaceuticals and functional foods. We consider that the concentrations of bioactive compounds in plants or fruits can be generally influenced by environmental factors, extraction methods, seasonality, biomass, and harvest time, among others. This information is essential for establishing future research niches and strengthening the relevance of using polyphenols in health. 

Ultimately, this research may propitiate new clinical and basic research that clarifies the relevance of polyphenol use in health and ameliorates the progression of the diseases mentioned in this particular issue.

## Data Availability

The data described here are publicly included in the special issue Polyphenols and Health Benefits: Volume I.
